# Root Functional Trait and Soil Microbial Coordination: Implications for Soil Respiration in Riparian Agroecosystems

**DOI:** 10.3389/fpls.2021.681113

**Published:** 2021-07-08

**Authors:** Kira A. Borden, Tolulope G. Mafa-Attoye, Kari E. Dunfield, Naresh V. Thevathasan, Andrew M. Gordon, Marney E. Isaac

**Affiliations:** ^1^Faculty of Land and Food Systems, The University of British Columbia, Vancouver, BC, Canada; ^2^Centre for Sustainable Food Systems, The University of British Columbia, Vancouver, BC, Canada; ^3^Department of Physical and Environmental Sciences, University of Toronto Scarborough, Toronto, ON, Canada; ^4^School of Environmental Sciences, University of Guelph, Guelph, ON, Canada

**Keywords:** absorptive roots, autotrophic respiration, heterotrophic respiration, plant functional traits, root economics spectrum, rhizosphere

## Abstract

Predicting respiration from roots and soil microbes is important in agricultural landscapes where net flux of carbon from the soil to the atmosphere is of large concern. Yet, in riparian agroecosystems that buffer aquatic environments from agricultural fields, little is known on the differential contribution of CO_2_ sources nor the systematic patterns in root and microbial communities that relate to these emissions. We deployed a field-based root exclusion experiment to measure heterotrophic and autotrophic-rhizospheric respiration across riparian buffer types in an agricultural landscape in southern Ontario, Canada. We paired bi-weekly measurements of in-field CO_2_ flux with analysis of soil properties and fine root functional traits. We quantified soil microbial community structure using qPCR to estimate bacterial and fungal abundance and characterized microbial diversity using high-throughput sequencing. Mean daytime total soil respiration rates in the growing season were 186.1 ± 26.7, 188.7 ± 23.0, 278.6 ± 30.0, and 503.4 ± 31.3 mg CO_2_-C m^–2^ h^–1^ in remnant coniferous and mixed forest, and rehabilitated forest and grass buffers, respectively. Contributions of autotrophic-rhizospheric respiration to total soil CO_2_ fluxes ranged widely between 14 and 63% across the buffers. Covariation in root traits aligned roots of higher specific root length and nitrogen content with higher specific root respiration rates, while microbial abundance in rhizosphere soil coorindated with roots that were thicker in diameter and higher in carbon to nitrogen ratio. Variation in autotrophic-rhizospheric respiration on a soil area basis was explained by soil temperature, fine root length density, and covariation in root traits. Heterotrophic respiration was strongly explained by soil moisture, temperature, and soil carbon, while multiple factor analysis revealed a positive correlation with soil microbial diversity. This is a first in-field study to quantify root and soil respiration in relation to trade-offs in root trait expression and to determine interactions between root traits and soil microbial community structure to predict soil respiration.

## Introduction

Soil respiration is a massive source of atmospheric carbon dioxide (CO_2_) and agricultural soils have been identified as a major contributor to global warming (Lal, 2003; Schlesinger and Andrews, 2000). Therefore, agricultural land use practices that mitigate and/or reverse net carbon (C) loss from soil need to be increasingly adopted. One such land use practice is the protection and restoration of riparian agroecosystems with trees (i.e., agroforestry) and/or perennial grasses (Tufekcioglu et al., 2003; Thevathasan et al., 2012; Udawatta and Jose, 2012). These “riparian buffers” are well understood in their importance to protect streambanks from erosion, capture nutrient run-off and leaching from adjacent cropping systems, and support biodiversity (Thevathasan et al., 2012; Christen and Dalgaard, 2013). Additionally, these specialized ecosystems can increase C storage in living tree and perennial biomass as well as in soil (Tufekcioglu et al., 2003; Fortier et al., 2013; Oelbermann et al., 2014). However, soil respiration in riparian systems is extremely variable (Tufekcioglu et al., 2001, 1999; Oelbermann et al., 2014; De Carlo et al., 2019), which makes it challenging to fully account for the net ecosystem exchange of C under various buffer types, and the associated plant community transformations, within agricultural landscapes.

Plant roots and soil microbes have complex relationships and interactions that drive their contributions to soil respiration (De Vries et al., 2016; Fry et al., 2019). Autotrophic-rhizospheric respiration (*R*_*a+r*_) is a function of roots respiring (root metabolic processes; i.e., autotrophic respiration) and the stimulated microbial activity in the rhizosphere (i.e., rhizospheric respiration) (Bardgett, 2017; Chen et al., 2019). Autotrophic-rhizospheric respiration can range dramatically from 10 to 90% of total soil respiration in vegetated ecosystems (Hanson et al., 2000). On the other hand, heterotrophic respiration (*R*_*h*_) from microbial decomposition of soil organic matter (SOM) can be both independent of plant root function (Ferlian et al., 2017) but also controlled by the quantity and quality of SOM from above and belowground litter inputs (De Long et al., 2019). Thus, roots can have both immediate effects on production of CO_2_ via *R*_*a+r*_ and longer-term effects via root turnover and contributions to SOM pools.

Trait-based plant ecology strives to explain plants’ response to the environment (“response”) and/or plants’ impact on the environment (“effect”) (Lavorel and Garnier, 2002; Violle et al., 2007). This approach has provided major advances in relating key plant traits to biogeochemical processes (De Vries et al., 2016; Borden et al., 2019; Coleman et al., 2020). The strength and importance of root traits in describing CO_2_ fluxes from soil is uncertain given the limited information from natural plant communities (De Long et al., 2019). However, general trends do show trade-offs in root trait expression, with specific root length (SRL) and root nitrogen content (N_*root*_) being positively related with root respiration and decomposition, while root diameter (D) and carbon to nitrogen ratio (C:N_*root*_) are positively related with root longevity (Sun and Mao, 2011; McCormack et al., 2015; Roumet et al., 2016; Ma et al., 2018). Thus, such covariation among root traits characterize the constraints in root construction and function across and within plant species (Kong et al., 2014; Prieto et al., 2015; Roumet et al., 2016; Borden and Isaac, 2019). Additionally, these root trait trade-offs may be related to microbial communities, with described relationships of species that form mycorrhizal fungi associations tending to have higher D and lower SRL (Ma et al., 2018; Bergmann et al., 2020). Concurrently, the expanding analytics of the soil microbiome has provided detail and range in capturing the composition of soil microorganisms (Toju et al., 2018). Taken together, these research advancements offer opportunities to systematically integrate plant traits, microbial communities, and agroecosystem processes (Pommier et al., 2018; Isaac and Borden, 2019; Fulthorpe et al., 2020). Yet, these belowground interactions have not been empirically related to respiration rates.

Our study was designed to determine (1) root trait covariation with root respiration rates, (2) root trait covariation with microbial communities, and (3) the subsequent effects of root traits and soil microbial richness and evenness on soil CO_2_ emissions in riparian agroecosystems. To do this, we performed a root exclusion experiment in four riparian buffer types (rehabilitated forest and grass buffers and remnant coniferous and mixed forest buffers) characterized by distinct plant communities in order to quantify the components of soil respiration: heterotrophic and autotrophic-rhizospheric respiration. We hypothesize that fundamental trade-offs in root construction and function coordinate with microbial community variation. We also expect that trait covariation, along with some key abiotic soil conditions, is an important explanatory variable for soil respiration.

## Materials and Methods

### Study Sites

We carried out a 10-week (May–August 2018) field experiment along Washington Creek, Ontario, Canada (43°18′N 80°33′W). The creek system is at an elevation of ∼300 m and has a mean annual temperature of 7.3°C and mean annual precipitation of 784 mm (1981–2010 Station Data; Environment Canada 2019). The creek is spring fed and situated in a region primarily under intensive agriculture of corn-soybean rotations. Soils are loam and classified as Gray Brown Luvisol with parent material composed of glacial till over limestone bedrock (Oelbermann et al., 2014). We maximized the potential range of belowground processes within the same creek system by sampling sites of distinct vegetative communities and variable soil properties. Four riparian buffer types were selected representing different perennial compositions. These buffers were either on sites with a history of managed rehabilitation (>30 years old), or on sites of remnant, old-growth forest: (i) grass and (ii) hardwood forest (referred to hereafter as “rehabilitated forest”) on rehabilitated land, and (iii) coniferous forest, and (iv) mixed forest on remnant land. The creek is alkaline (7.5–8.5) (Oelbermann et al., 2014) and soil pH at these sites ranged from 7.1 to 7.6, with higher values at the younger, rehabilitated grass and forest sites (7.5 and 7.6, respectively) compared to the old-growth forest sites (7.1). Soil inorganic C was also higher at the rehabilitated buffers (8 and 11%) (unpublished data; Supplementary Table 1). For more details on study sites refer to De Carlo et al. (2019), Oelbermann et al. (2014), and Mafa-Attoye et al. (2020).

### Soil Respiration

We used the root exclusion method (i.e., difference method) to estimate autotrophic + rhizospheric respiration (*R*_*a+r*_) and differentiate CO_2_ from heterotrophic respiration (*R*_*h*_) in soil (Hanson et al., 2000; Lavigne et al., 2003; Kuzyakov and Larionova, 2005). We did so in a nested sampling design: four sampling plots of 1.0 × 0.5 m were established within each buffer type. In each sampling plot, root exclusion sub-plots were created in half the area (0.5 × 0.5 m) and trenched to 40 cm. Soil within trenched sub-plots was carefully removed and living roots were removed from that soil. Landscaping fabric was inserted to line the exclusion plots to prevent root growth into the exclusion area and soil was gently returned to the exclusion area, with effort made to replace soil at the same depth and with similar compaction (Lavigne et al., 2003). Soil collars were inserted in the center of each section, 2.5 cm into the soil. In the root-exclusion sub-plots, newly established plants were removed by hand throughout the experiment. At the end of the experiment, after digging out the landscaping fabric, we observed roots had not penetrated through the fabric. At each sampling time, any physical disturbance like trenching or adjustment of soil collars was carried out after CO_2_ measurements were taken.

Root exclusion experiments have limitations due to possible effects on soil conditions from soil disturbance and lack of vegetative cover (Hanson et al., 2000; Kuzyakov and Larionova, 2005). Presumably, an increase in respiration from soils occurred immediately after soil disturbance particularly during exclusion plot set-up, but effort was made to protect soil during removal and replacement, to minimize disturbance of soil aggregates, and then during the two-week stabilization period prior to the first measurements of soil respiration. We tested our assumption of similar soil conditions by comparing bulk soil bacteria and fungi abundance, soil moisture, and soil temperature between paired inclusion and exclusion sub-plots on a subset of sampling dates, which is further explained in the statistical analysis section.

Every 2 weeks following soil collar installation, soil CO_2_ flux was measured using a portable infrared gas analyzer (Licor L6400XT) with a soil CO_2_ flux closed chamber. Measurements across all sites were completed on the same day between 09:00 and 14:00 with the order of buffer type and sampling plot randomized. Ambient CO_2_ near the soil surface was measured prior to measurements and used to set target CO_2_ and range. The average of three cycles per soil collar was used to calculate CO_2_ flux. Total soil respiration (*R*_*s*_; μmol CO_2_ m^–2^ s^–1^) was measured in the root-inclusion sub-plots, while fluxes of CO_2_ from root-exclusion sub-plots captured heterotrophic respiration (*R*_*h*_; μmol CO_2_ m^–2^ s^–1^). Autotrophic + rhizospheric respiration (*R*_*a+r*_; μmol CO_2_ m^–2^ s^–1^) was calculated as the difference between *R*_*s*_ and *R*_*h*_.

### Soil Physico-Chemical Properties

Soil temperature was measured using a temperature probe (LI-COR #6000-09TC) inserted to a depth of ∼15 cm near the soil collar at each sampling time. From each plot, soil and root samples (explained in the following section) were collected using a soil corer of known volume (100 cm^3^) in the top 10 cm of soil within root inclusion areas. Soil moisture was determined on ∼5 g of field moist soil, dried at 105°C for 48 h, to calculate gravimetric content. Available nitrate (NO_3_^–^) and available ammonium (NH_4_^+^) in 1:10 field fresh soil to KCl solution extractions were measured colormetrically on a flow injection analyzer (QuikChem8500; Lachat Instruments, Milwaukee, WI, United States). Another subsample of soil was dried and ground and analyzed for total C and N with an elemental analyzer (CN 628, LECO Instruments, Mississauga, ON, Canada). All soil chemical analyses were completed at University of Toronto Scarborough.

### Root Sampling and Analysis

At the start of the experiment, fine roots were removed from root-exclusion sub-plots to sample root traits and corresponding microbial abundance in rhizosphere soil, which is explained in the microbial sampling sections. Over the course of the experiment, and at the time of each soil respiration sampling date, roots were extracted from 100 cm^3^ soil cores collected from the 0–10 cm soil depth. After soil was sub-sampled for soil properties, roots were extracted by washing samples over sieves, further cleaned to remove adhering soil particles, and then processed for further analysis. We focused our analysis on absorptive fine roots, which are most responsible for nutrient uptake and have the highest respiration rates (McCormack et al., 2015), and excluded rhizomes collected from grasses and “transport” roots from woody vegetation (see McCormack et al., 2015; Borden et al., 2020). Image analysis of root morphology (total length and average diameter) was measured using WinRhizo 2019a (Reagent Instruments Inc., Canada), and then standardized using the dry weight biomass of root samples after 48 h at 60°C. Dried root samples were then ground and analyzed for total C and N using an elemental analyzer (CN 628, LECO Instruments, Mississauga, ON, Canada).

With root length data from each sampling volume, we calculated fine root length density (FRLD; cm cm^–3^), which excluded rhizomes and root orders >3 of woody plants. At the scale of individual roots, we measured traits that are positively associated with resource acquisition: specific root length (SRL; m g^–1^), and root nitrogen content (N_*root*_; mg g^–1^); and root traits positively associated with root tissue longevity: average root diameter (D; mm), and root C to N ratio (C:N_*root*_). We also calculated the specific root respiration (*R*_*root*_; nmol CO_2_ g^–1^ s^–1^), by dividing *R*_*a+r*_ respiration by the absorptive fine root biomass of that same plot on the same sampling date, as a standardized indicator of root-rhizosphere activity (Makita et al., 2012; Roumet et al., 2016; Zhou et al., 2018).

### Microbial Sampling and Analysis

Microbial sampling and analysis occurred separately for soil adhering to roots (rhizosphere) and bulk soil. Rhizosphere soil (DNA) was analyzed for targeted genes to quantify bacteria and fungi abundance. Bulk soil (cDNA) was further analyzed for potential activity of targeted transcripts of bacteria and fungi, and sequenced in order calculate microbial diversity. Methods for both are described below and a summary of sampling, processing, and analysis steps is provided in Supplementary Table 2.

#### Microbial Sampling

##### Rhizosphere soil

For microbial analysis of rhizosphere soil, we collected all roots including attached soil (after shaking) that were exhumed from each root exclusion sub-plot. In the lab, absorptive roots were homogenized and subsampled into three batches from each plot. Rhizosphere soil was separated from the roots using the methods described previously by Donn et al. (2014). The adhering soil and the root samples were vortexed thrice for 30 s each time, and 20 mL from the rhizosphere soil mixture obtained was centrifuged at 5000 r.p.m for 15 min at 5°C. The resulting pellets were stored at −20°C prior to DNA extraction. Roots were rinsed and stored at 4°C until processing, as described in section “Root Sampling and Analysis.”

##### Bulk soil

For microbial analysis of bulk soil, bi-weekly soil samples were collected from both root exclusion and root inclusion sub-plots on the same day that soil respiration, soil, and roots were sampled. Three random soil samples were collected from 0–10 cm depth, gently homogenized, and ∼2 g of composited sample was immediately transferred into pre-weighed sterile tubes containing 3 mL of LifeGuard soil preservation solution (MO BIO Laboratories, Inc., Carlsbad, CA, United States) to stabilize the RNA. Tubes were stored and transported on ice, then transferred to −80°C freezer. Based on soil respiration data we selected two dates for intensive sequencing analysis. We chose dates that showed (i) large CO_2_ emission rates representative of the peak of vegetative growth (July 4) and (ii) later in the summer when emissions remained high and stabilized (August 15).

#### Nucleic Acid Extraction and Quantitative Real-Time PCR

From rhizosphere soil, DNA was extracted using the PowerSoil DNA Isolation Kit (QiagenR Valencia, CA, United States) following the manufacturer’s protocol. From bulk soil samples, RNA and DNA were co-extracted using RNeasy PowerSoil^TM^ Total RNA Kit and DNA Elution Kit (Qiagen^®^, Valencia, CA, United States) following the manufacturer’s instructions. The RNA obtained was subjected to DNase treatment and then reverse transcribed to complementary DNA (cDNA) suitable for qPCR.

The total bacterial (16S rRNA) and fungal (18S rRNA) genes and transcripts from rhizosphere and bulk soil, respectively, were quantified by performing qPCR. Primer pairs 338F/518R (16S rRNA; Fierer et al., 2005) and FF390/FR1 (18S rRNA; Vainio and Hantula, 2000) were used for target genes and transcripts (further details provided with Supplementary Table 3). Samples were analyzed in duplicates in 96-well PCR plates with a Bio-Rad CFX detection system (Bio-Rad Laboratories, Inc., Hercules, CA, United States). The PCR efficiency, R^2^, and slope of the standard curve for quantification were 16S (101.9%, 0.99, and −3.27), 18S (99.2%, 0.98, and −3.34).

#### Sequencing and Bioinformatics of Bulk Soil Microbial Communities

High-throughput Illumina MiSeq sequencing approach was used to quantify the diversity of bacterial and fungal communities from bulk soil in root inclusion sub-plots. The extracted cDNA was sent to McGill University and Génome Québec Innovation Center, Montréal (Québec) Canada, and analyzed via the Illumina MiSeq platform (Illumina Inc., San Diego, CA, United States) using 515F/806R (bacteria) and ITS1F/ITS2 (fungi) primer sets (White et al., 1990; Gardes and Bruns, 1993; Apprill et al., 2015; Parada et al., 2016). Sequenced data from Illumina fastq files for bacterial 16S rRNA and fungal ITS transcripts were processed and analyzed using Quantitative Insights Into Microbial Ecology (QIIME 2, version 2019.1). The SILVA-132 and UNITE databases were used to assign taxonomy to the amplicon sequence variants (ASVs) for bacteria and fungi, respectively. Alpha diversity metrics such as Shannon’s diversity index, Observed ASVs, and Faith’s Phylogenetic Diversity and Pielou’s evenness were computed from QIIME.

### Statistical Analysis

All statistical analyses were completed in R 3.5.0 (R Core Team., 2019). Our analyzed dataset from four riparian buffer types with four sampling plots each over eight sampling times includes *n* = 120 soil, root data and respiration measurements, *n* = 38 bulk soil microbial data, as well as an initial collection of *n* = 14 paired microbial rhizosphere soil and root trait data. One plot in the rehabilitated forest was omitted due to concern of effects from streambank erosion. Parametric assumptions of normality were evaluated visually and using the Shapiro-Wilk test. When necessary, variables were square root (*R*_*s*_, *R*_*h*_, *R*_*a+r*_, FRLD, soil available N) or log transformed (roots: SRL, N_*root*_, D, C:N_*root*_, *R*_*root*_; soil: moisture, temperature, C, C:N; microbial: 16S, 18S).

To characterize the range of soil physico-chemical properties among the buffer types, variable means are presented in Supplementary Table 1. Cumulative daytime CO_2_ emissions over an 84-day period (Mg CO_2_-C ha^–2^) were calculated by linear interpolation of the bi-weekly measurements from June 6 and August 29 using the *gasfluxes* package (Fuss, 2019) and differences among the buffer types were evaluated using ANOVA. We also evaluated the assumptions that establishment of root-exclusion sub-plots had minimal effect on abiotic and biotic conditions in soil during our experiment by using paired t-tests on soil moisture, soil temperature, abundance and activity of bacteria and fungi communities (16S and 18S genes and transcripts) between paired inclusion and exclusion sub-plots on the same sampling date.

Using our data set of root traits paired with soil respiration measurements, we evaluated how *R*_*root*_ covaries with root traits in a principal component analysis (PCA) using the *vegan* package (Oksanen et al., 2019). This approach quantifies the strength of covariation and trade-offs in root trait expression on the dominant PCA axes. We evaluated the influence of the type of riparian buffer and sampling date, and their interaction, on coordinated root trait expression (PCA axis scores) using two-way ANOVA. We also assessed overall root trait covariation with microbial abundance in a second PCA using our dataset containing paired root traits and bacteria and fungi abundances in rhizosphere soil.

We assessed drivers of soil respiration rates in riparian buffers using two approaches. In our first approach using the full data set, linear mixed models (LMM) fitted with REML in the *nmle* package (Pinheiro et al., 2018) quantified how soil and root variables explained *R*_*s*_ and its components: *R*_*h*_ and *R*_*a+r*_. Fixed effects were selected *a priori* based on known dominant controlling factors for microbial and/or root respiration: soil moisture, soil temperature, available N in soil (NO_3_^–^ and NH_4_^+^), soil C, soil C:N, rooting density (FRLD), and we also included overall absorptive root syndromes by using PCA axes scores. We treated sampling plot nested in riparian buffer type as a repeated measure assigned as a random effect. We then assessed the proportion of variance explained by the continuous soil and root variables alone (marginal *r*^2^) versus when random effects of riparian buffer and sampling plot are included (conditional *r*^2^) (Nakagawa and Schielzeth, 2013). The proportions of variation explained by included random effects were estimated using variance decomposition with the *ape* package (Paradis et al., 2004). In our second approach, using the subset of data from two sampling dates with observations of microbial community structure in bulk soil, we used multiple factor analysis (MFA) to assess how groups of variables: microbial abundances, microbial diversity indices, root traits, and soil properties, covary together or independently with each other and with *R*_*h*_. Riparian buffer type and sampling date were assigned as supplementary variables. Basically, in this analysis PCA is performed first separately for each group of variables, then the resulting standardized group-based PCA data are used to perform an overall PCA to evaluate how the groups of variables covary. We evaluated group similarity (correlation) by calculating the RV coefficient and performing a Monte Carlo permutation (*n* = 1000) to test if group correlations were significantly different than when randomly generated. For MFA, we used the “*FactoMineR”* (Le et al., 2008) and “*ade4*” (Dray and Dufour, 2007) packages.

## Results

### Total, Heterotrophic, and Autotrophic-Rhizospheric Respiration

**FIGURE 1:**
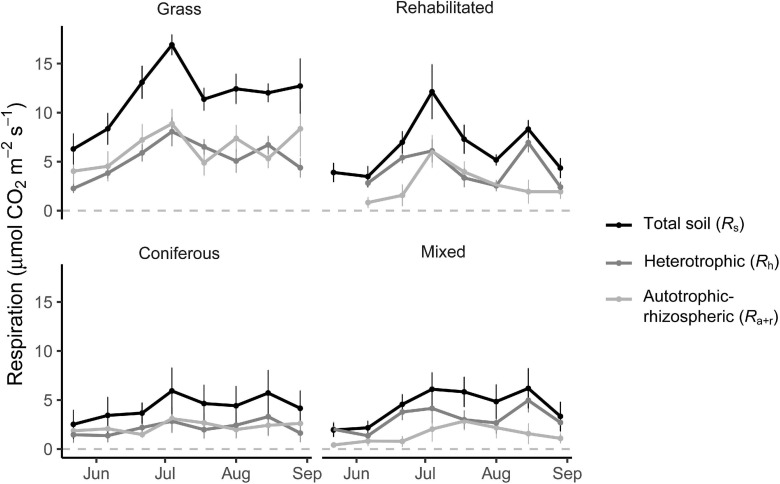
Soil respiration rates (μmol CO_2_ m^– 2^ s^– 1^) measured from root inclusion and exclusion sub-plots. Data shown are means ± SE of sampling plots (*n* = 4) from each land use.

Soil respiration rates fluctuated over the growing season in the grass buffer and rehabilitated forest and were generally above that of coniferous forest and mixed forest (Figure 1). Average daytime total soil respiration rates were 186.1 ± 26.7, 188.7 ± 23.0, 278.6 ± 30.0, and 503.4 ± 31.3 mg CO_2_-C m^–2^ h^–1^ in the coniferous, mixed, rehabilitated forest, and grass buffer, respectively. Percent contributions of *R*_*a+r*_ to *R*_*s*_ ranged from 14 to 63% and was proportionally high in the grass buffer and coniferous forest >40% (Figure 1). Estimated cumulative *R*_*h*_ emissions from daytime measurements ranged between 2.1 ± 2.0 (coniferous forest) to 5.3 ± 1.6 (grass) Mg CO_2_-C ha^–1^ and *R*_*a+r*_ emissions ranged between 1.5 ± 1.5 (mixed forest) to 5.8 ± 2.2 (grass) Mg CO_2_-C ha^–1^ (Table 1). There were significant differences among riparian buffer types for *R*_*s*_ emissions (*p* = 0.009) and *R*_*a+r*_ emissions (*p* = 0.01) but not for *R*_*h*_ emissions (*p* = 0.09) (Table 1). Grass buffer had significantly higher *R*_*a+r*_ emissions and corresponding *R*_*s*_ emissions compared to coniferous forest and mixed forest but had non-significant (*p* = 0.13) and marginally significant (*p* = 0.09) differences from rehabilitated forest in *R*_*s*_ and *R*_*a+r*_ emissions, respectively (Table 1). There were no significant differences in soil moisture, soil temperature, and microbial (16S and 18S) activity or abundance between paired root exclusion and inclusion sub-plots, except for 0.5°C higher soil temperature in exclusion sub-plots on July 4 (*p* < 0.01) (Supplementary Figure 1).

**TABLE 1:** Cumulative soil CO_2_ emissions (Mg C ha^–1^) between June and August 2018 (84-day sampling period) estimated from measured daytime emissions.

**Buffer type**	**Heterotrophic (*R*_*h*_)**	**Autotrophic-rhizospheric (*R*_*a+r*_)**	**Total (*R*_*s*_)**
Grass	5.72 ± 0.87	6.46 ± 1.35 a	12.18 ± 1.25 a
Rehabilitated	3.89 ± 0.53	2.51 ± 0.51 ab	6.88 ± 1.10 ab
Coniferous	2.24 ± 1.09	2.29 ± 0.83 b	4.54 ± 2.00 b
Mixed	3.19 ± 0.96	1.57 ± 0.78 b	4.69 ± 1.39 b

*Same letters indicate no significant differences in respiration component between riparian buffer types.*

### Covariation of Root Traits and Microbial Communities

**FIGURE 2:**
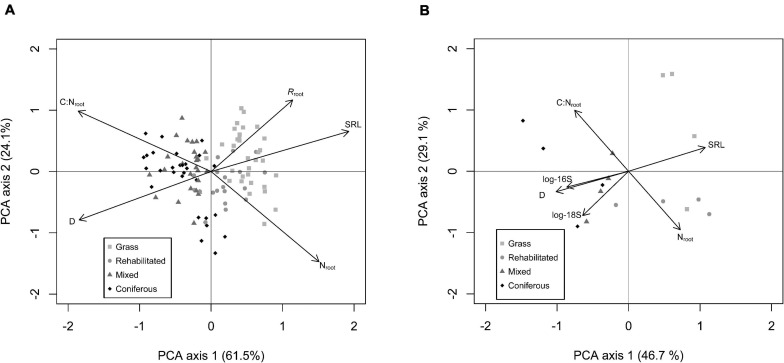
Principal component analysis (PCA) of absorptive fine root traits: D = average root diameter (mm); C:N_*root*_ = root C to N ratio; SRL = specific root length (m g^– 1^); N_*root*_ = root N content (mg g^– 1^); *R*_*root*_ = specific root-rhizosphere respiration (nmol CO_2_ g^– 1^ s^– 1^) from full data set (*n* = 120) **(A)**. PCA of absorptive fine root traits measured with paired rhizosphere soil analysis of bacterial (16S) and fungal (18S) abundance **(B)**.

Covariation in absorptive fine root traits was well explained by the first principal component axis (∼50 to 60% of total covariation) both for our full-season trait data set with respiration rates (Figure 2A) as well as the analysis of roots and microbial communities in rhizosphere soil (Figure 2B). On this axis, roots expressing higher SRL and N_*root*_ also had higher *R*_*root*_, and were in opposition to roots expressing thicker D, higher C:N_*root*_. All root traits were significantly correlated with PC1 (Supplementary Table 4). In rhizosphere soil, PCA of root traits and rhizosphere microbial communities indicate that higher abundances of 16S and 18S coordinated with thicker D and higher C:N_*root*_ on PC1 (Figure 2B). PC2 explained >20% of total covariation (Figure 2) and in the respiration data set, shows a trade off in roots with higher SRL, C:N_*root*_, and *R*_*root*_ opposed to roots that have thicker D and higher N_*root*_. For roots with microbial abundance measured in the rhizosphere, C:N_*root*_ was strongly featured on PC2 in a trade-off with N_*ab*_ and 18S (Figure 2B; Supplementary Table 4).

The type of riparian buffer was important in controlling the relative position of individual observations on PC1 (*F*_3_,_99_ = 82.72; *p* < 0.001) with roots in grass > rehabilitated forest > mixed forest > coniferous forest having relatively higher PC1 scores (i.e., higher SRL, N, and *R*_*root*_). Date was also significant (*F*_1_, _99_ = 3.25; *p* = 0.07) with PC1 scores shifting lower (i.e., thicker D, higher C:N_*root*_, and lower *R*_*root*_) later in the summer compared to earlier sampling dates. There was no interaction of buffer type × date on PC1 scores (*F*_3_,_99_ = 2.56; *p* = 0.08). For PC2 scores, there were significant effects from buffer type (*F*_3_,_99_ = 6.96; *p* < 0.001), with PC2 scores relatively higher for grass > mixed > coniferous > rehabilitated forest. Broadly, PC2 scores increased (i.e., higher SRL, C:N_*root*_, and *R*_*root*_) over the season, with a significant main effect of date (*F*_1_,_99_ = 20.02; *p* < 0.001) but the extent of this increase over time depended on buffer type, with a significant buffer type × date interaction (*F*_3_,_99_ = 6.85; *p* < 0.001).

### Abiotic and Biotic Soil Environment Variables in Relation to Soil Respiration

Our experiment occurred over a range of soil abiotic soil conditions in the study sites on the same creek system (Supplementary Table 1). In explaining *R*_*s*_ and its components (*R*_*h*_ and *R*_*a+r*_), soil temperature was consistently a significant positive predictor (Table 2). Soil moisture was a negative predictor of *R*_*s*_ and *R*_*h*_, while available NH_4_^+^ was a negative coefficient for *R*_*s*_ and soil C was a positive coefficient of *R*_*h*_. For *R*_*a+r*_ the only significant explanatory variables other than soil temperature were root variables: positive FRLD and positive PC1 axis scores and PC2 axis scores associated with higher *R*_*root*_ (Table 2). Fixed effect variables explained 49 to 61% of variation in soil respiration and its components, and when also accounting for random effects (i.e., non-measured effects of inherent differences among the riparian buffer types, or among individual sampling plots within each buffer type) improved the explained variance to between 75 and 86% (Table 2). Variance decomposition showed the repeated measure on the sampling plots nested in buffer type contributed the most to the improvement in explained variance, by 20.4, 16.0, and 16.2% for *R*_*s*_, *R*_*h*_, and *R*_*a+r*_, respectively, while buffer type (i.e., other site level differences that were not measured and included as fixed effects) contributed only 2.4, 0.3, and 0.9% for *R*_*s*_, *R*_*h*_, and *R*_*a+r*_, respectively.

**TABLE 2:** Coefficients of linear mixed models to predict total soil respiration (*R*_*s*_), heterotrophic respiration (*R*_*h*_), and autotrophic-rhizospheric respiration (*R*_*a+r*_).

		**Soil variables**						**Root variables**				
Soil respiration component	Intercept	log-soil moist.	log-soil temp.	sqrt-soil NO_3_^–^	sqrt-soil NH_4_^+^	log-soil C	log-soil C:N	sqrt-FRLD_*ab*_	PC1	PC2	Marginal *r*^2^	Conditional *r*^2^
*R*s	−1.905	−**1.463**	**0.064**	0.031	−**0.052**	0.629	1.521	0.121	**0.488**	**0.438**	0.61	0.86
*R*_*h*_	−2.288	−**1.906**	**0.044**	0.015	−0.024	**1.364**	0.294	0.017	0.268	0.117	0.50	0.82
*R*_*a+r*_	−2.474	−0.336	**0.044**	0.032	−0.049	−0.146	2.966	**0.178**	**0.394**	**0.576**	0.49	0.75

*Coefficients of fitted fixed effects are reported with significant coefficients in bold. Total variation explained by fixed effects of soil and root variables (marginal *r*^2^) and when random effects (sampling plot nested in land use) are included (conditional *r*^2^) are shown.*

**FIGURE 3:**
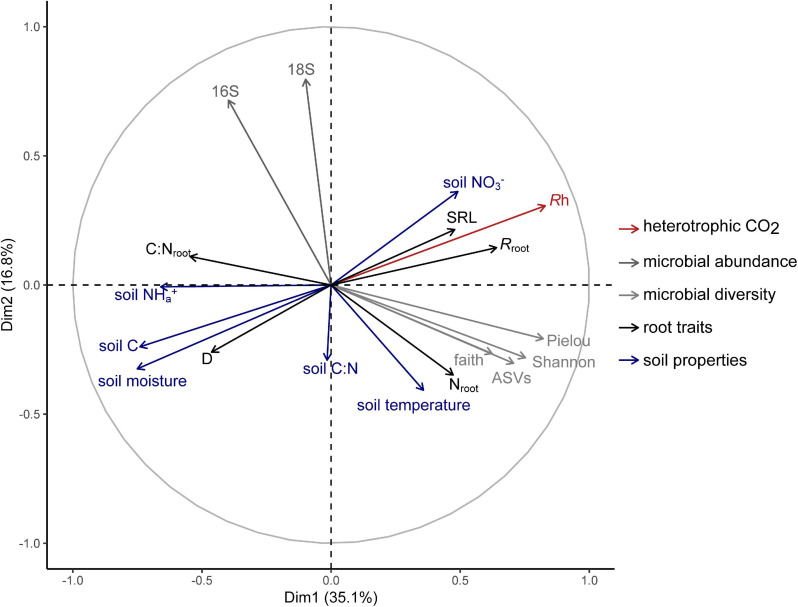
Multiple factor analysis of heterotrophic respiration (*R_h_*) and grouped biotic and abiotic soil environment variables: microbial abundance [bacterial (16S) and fungal (18S) abundance]; microbial diversity [Shannon’s diversity index, Observed ASVs, Faith’s Phylogenetic Diversity, and Pielou’s evenness]; root traits [average root diameter (D), root C to N ratio (C:N,root), specific root length (SRL), root N content (*N*_*root*_), and specific root-rhizosphere respiration (*R*_*root*_)]; and soil properties [soil moisture, soil temperature, total soil C, soil C:N, available NO_3_^–^, and available NH_4_^+^].

Multiple factor analysis revealed grouped variables: root traits and soil properties were significantly correlated (RV = 0.32; Table 3), such that roots with thicker D and higher C:N_*root*_ were aligned with soil high in moisture, available NH_4_^+^, and soil C, while roots with higher SRL, N_*root*_, and *R*_*root*_ were associated with soil with higher available NO_3_^–^ (Figure 3). Heterotrophic respiration was related to covariation in soil properties (RV = 0.38) and less so to covariation in root traits (RV = 0.12) (Table 3). We observed a range of microbial diversity in bulk soil, with significantly higher diversity in the rehabilitated forest buffer than in the coniferous forest buffer (Supplementary Table 5). However, there was no significant difference in microbial abundances among buffer types (Supplementary Figure 2). In MFA, microbial diversity indices were collectively positively correlated with *R*_*h*_ (RV = 0.35), while microbial abundance was not (RV = 0.04) (Table 3). Microbial abundance and diversity in bulk soil were independent to root trait covariation (Figure 3 and Table 3).

**TABLE 3:** RV coefficients (top right of table) between groups of abiotic and biotic environment variables and heterotrophic respiration (*R*_*h*_) from multiple factor analysis (Figure 3).

	***R*_*h*_**	**Soil properties**	**Microbial abundance**	**Microbial diversity**	**Root traits**
*R*_*h*_	–	**0.38**	0.04	**0.35**	**0.12**
Soil properties	0.005	–	0.08	0.18	**0.32**
Mic. abundance	0.279	0.202	–	0.14	0.04
Mic. diversity	0.001	0.104	0.679	–	0.10
Root traits	0.031	0.002	0.670	0.062	–

*Coefficients are in bold if they are significantly (*p* < 0.05) correlated based on simulated *p*-values from a Monte Carlo test (bottom left of table).*

## Discussion

### Coordination Belowground? Root Traits and Microbial Communities

Our results show for the first time, to our knowledge, a dominant root trait axis inclusive of *R*_*root*_. Specific root respiration was associated with roots expressing higher SRL and N_*root*_ (Figure 2A). This observed root trait covariation support a hypothesized “root economics spectrum” (Kong et al., 2014; Roumet et al., 2016; Borden et al., 2020) related to the C economy in plants (Roumet et al., 2016).

Bacteria and fungi abundance in rhizosphere soil were also aligned with root trait covariation, associating with roots of thicker D and lower SRL, and for fungi abundance, roots with thicker D and higher N_*root*_ (Figure 2B). These results follow previously identified trade-offs in resource acquisition strategies on a root-microbe continuum (Ma et al., 2018; Bergmann et al., 2020) [i.e., when resource acquisition is “outsourced” to associated fungi (Bergmann et al., 2020)]. Conversely, in bulk soil, root trait covariation seemed unrelated to microbial activity (Figure 3). Other studies report mixed results. For instance, De Long et al. (2019) observed that higher root N was positively related with higher fungal and bacterial abundances in bulk soil, while Leff et al. (2018), found no relationships between root traits and bacteria or fungi community composition in bulk soil. In this study, it appears that covariation of soil microbial abundances in rhizosphere soil with root traits may not be due to the underlying microbial abundances in bulk soil, with potential differences in microbial communities between rhizosphere soil and bulk soil (Donn et al., 2014).

We observed generalized shifts in root traits among riparian buffers and sampling dates (Figure 2A). While our study was designed to examine community-level root trait variation, it is likely that not only species composition but also phenological variation and/or intraspecific variation may also have an effect on expression of root traits. For example, nutrient gradients can shift root morphology and anatomy within species (Wang et al., 2017; Borden and Isaac, 2019). Similarly, Donn et al. (2014) found that microbial community structure changed over time in rhizosphere but not in bulk soil, suggesting plant phenology-driven effects on the soil microbiome. As we observed a significant effect of sampling time on root trait expression, it could be expected that the rhizosphere microbial profile and relationship with root traits could vary over the growing season. Additional work is needed to test effects of seasonality.

### Disentangling Belowground Drivers of Soil Respiration

Respiration from heterotrophic and autotrophic processes in soil occur and vary at different spatial and temporal scales (Gomez-Casanovas et al., 2012; Jacinthe and Vidon, 2017). Our study and previous research show high heterogeneity of CO_2_ emissions and soil conditions within these riparian buffers (De Carlo et al., 2019). Over the growing season soil respiration varied and peaked in early July, and markedly so in the grass buffer (Figure 1), driven by the high density of roots. Steinauer et al. (2017) found that denser root systems (i.e., amount of roots in soil) and shallower root system (i.e., location of roots in soil) resulted in higher total soil respiration in grasslands. In our study, not only the density of roots in soil but the expressed root traits led to higher *R*_*a+r*_ (Table 2). Thus, organ-level variation in roots may also have a predictable impact on autotroph-rhizosphere respiration measured on a soil area basis, and these patterns may influence CO_2_ emissions from soil over a growing season. Broadly, our findings of systematic variation of root traits in predicting respiration rates within and across the studied buffer types contribute to identifying empirical relationships that are meaningful for ecosystem-level analysis across a range of scales and riparian agroecosystems. Notably, root trait covariation remained important predictors of total soil respiration, thus supporting root functional trait integration into biogeochemical models of total soil respiration.

Root respiration is thought to correlate positively with root decomposition rates (Prieto et al., 2016; Roumet et al., 2016). Thus, it would be expected the root traits will relate to how root litter inputs affect heterotrophic respiration. However, in our study, evidence of root effects on soil processes was weak to negligible. Covariation in root traits showed a weak association with *R*_*h*_ from MFA (Table 3) and, when accounting for individual soil properties, was not a predictor of *R*_*h*_ in the linear mixed model (Table 2). A lack of association between root traits and microbial respiration in bulk soil could be due to the temporal lag between root turnover and decomposition of roots which is not captured in the time scale of our study. Similarly, Ferlian et al. (2017) found soil stoichiometry, rather than root stoichiometry, to be a main determinant of soil microbial biomass and total soil respiration from soil collected near roots of angiosperm trees. On the other hand, microbial diversity showed a stronger correlation with *R*_*h*_. Liu et al. (2020) found systematic trends in microbial communities in predicting total soil respiration rates across distinct rice paddies and corn-wheat cropping environments. A greater diversity of microorganisms in soil may enable decomposition of a wider range of soil organic matter, thus leading to overall higher rates of respiration from soil (Maron et al., 2018).

Biogeochemical models require identifying key abiotic controls of biological activity that collectively drive C exchange (Fry et al., 2019). The two major abiotic controls of soil respiration: moisture and temperature, which regulate biological activity and gas diffusion in soil, were strong explanatory variables of *R*_*h*_. Due to large variability in soil moisture in riparian systems within a buffer zone and over the growing season, soil hydrological conditions may be particularly important in controlling belowground respiration (Gordon et al., 1987; Jacinthe and Vidon, 2017). For root-derived respiration, soil temperature was an important control of *R*_*a+r*_, reflecting the importance of soil temperature on root growth and activity (Boone et al., 1998). While not empirically tested in this study, relatively high soil pH and soil inorganic C in the rehabilitated buffers compared to the remnant forest buffers, may also control site-level soil respiration rates. On larger soil pH gradients, heterotrophic respiration has been found to increase with higher soil pH, while root respiration decreases (Chen et al., 2016). Additionally, and particularly in the rehabilitated buffers, the proportion of CO_2_ emissions from soil originating from carbonates (soil inorganic C) could be significant, and CO_2_ from microbial respiration may react with carbonates in soil (Ramnarine et al., 2012). More detailed analysis of sources of CO_2_ emissions could reveal complex C exchanges in the soil profile at these sites.

During the timeframe of this field experiment, we assumed that there was negligible influence of root removal on the soil environment between inclusion and exclusion sub-plots, which validated calculated *R*_*a+r*_. From our comparative test on soil moisture and temperature and total microbial abundance and activity between paired sub-plots, this assumption held. The only exception was on one sampling date when soil temperature in the exclusion sub-plots were warmer than in paired inclusion sub-plots demonstrating the effects of vegetation on regulating soil temperature and considerations when carrying out root exclusion field studies. Shade cloth over exclusion sub-plots may improve the similarity between sub-plots particularly during a peak in summer temperatures and solar irradiance. Gentle removal and replacement of soil in the exclusion plots at matched depths was carried out to avoid disturbing microaggregates and maintain initial soil compaction as best as possible. However, bulk density may have changed, particularly in plots that had a high density of roots, and it is likely there was some disturbance to soil macro- and micro-porosity. Additionally, while total bacteria and fungi abundance and activity were similar between the inclusion and exclusion sub-plots, additional work is needed to understand potential effects on microbial community composition given our finding that *R_h* positively correlated with microbial diversity. Despite these possible sources of error, these destructive studies remain a viable method to differentiate heterotrophic from root-derived respiration in the field when isotopic analysis (i.e., ^13^C natural abundance or tracer methods) is not applicable or impractical for extensive field-deployed studies.

### Riparian Agroecosystem Management Implications

Riparian buffers can lessen the negative impacts of agricultural production on the aquatic environment, but less is known on greenhouse gas emissions to the atmosphere. Therefore, accurate estimates of CO_2_ emissions from riparian agroecosystems are necessary for assessing climate change mitigation versus warming potential. However, the diversity of management and vegetative structure and composition (i.e., in existing forest, new forest, or other perennial buffers) can be challenging to draw generalizable conclusions. In this regard, frameworks that can systematically measure and compare ecosystem processes across diverse riparian systems are useful. De Long et al. (2019) found that plant traits and soil properties improved predictions of C fluxes in the ecosystem (ecosystem respiration and net ecosystem exchange) in plant monocultures but not in mixed communities. In our study, we found evidence of coordinated belowground root-microbe strategies and correlation between root traits and autotrophic-rhizospheric respiration (i.e., root-derived respiration) and total soil respiration, suggesting measurable integration across trophic levels and an application to improve modeled C based on belowground plant traits.

Distinguishing sources of CO_2_ allows for more refined predictions of climate mitigation and CO_2_ emissions that differ in spatial and temporal scales. Soil respiration in temperate riparian systems is markedly highest during summer months (Oelbermann et al., 2014; De Carlo et al., 2019). Thus, the major differences in CO_2_ emissions among riparian buffer types were likely captured during the sampling period of the present study, although our results do not represent annual patterns. Differences of cumulative emissions based on daytime measurements among buffer types were accentuated by autotrophic-rhizospheric respiration, particularly in a grass buffer with high density of fine roots (Table 1 and Figure 1). Thus, in certain types of riparian buffers, root-derived respiration can substantially elevate total CO_2_ emissions from soil.

However, soil respiration indicates higher biological activity in soil and relates to other important ecological processes. In different studies at the same study sites, nitrous oxide (N_2_O) emissions were relatively low in the rehabilitated grass buffer, with observed differences in nitrifying bacteria community structure (Mafa-Attoye et al., 2020), while methane (CH_4_) emissions were starkly higher in remnant mixed forest buffer (Buchanan et al., 2020) where soil moisture levels were highest. Given that N_2_O and CH_4_ have, respectively, 298 and 25 times the warming potential as CO_2_, full assessment of greenhouse gases fluxes and their trade-offs are required to optimize riparian buffer management for climate change mitigation. In rehabilitating or maintaining existing riparian forests and grassland buffers, a refined understanding of sources of respiration and their interactions with key abiotic controls is essential for accurate estimates of net ecosystem exchange of C and as part of a full assessment of ecosystem services in the agricultural landscape.

## Conclusion

This is one of the first field studies to examine interactions between covariation of root traits and soil microbial community abundance and diversity to investigate sources of soil respiration. Our findings reveal strong covariation of root traits with specific root respiration rates and this coordination in root traits was important in predicting soil CO_2_ emissions on an area basis. We also show that root traits covaried with microbial abundance in rhizosphere soil but not in bulk soil, while microbial diversity in bulk soil was positively associated with heterotrophic respiration. As respiration from soil represents the major pathway of C transfer from the ecosystem to the atmosphere, strategic management and design of riparian buffers can begin to adopt functional trait approaches to assess the belowground processes that regulate delivery of key ecological services for climate change mitigation.

## Data Availability Statement

The raw sequence data are deposited in NCBI SRA under the project PRJNA718381. The non-sequencing data are deposited in Scholars Portal Dataverse: https://doi.org/10.5683/SP2/O1MKRS.

## Author Contributions

KB, MI, KD, TM-A, and NT conceived and planned the experiments. KB and TM-A carried out the experiments and analyzed the data. KB completed the statistical analysis and wrote the manuscript. MI, KD, NT, and AG managed project funding and provided resources to complete the research. All authors provided critical feedback and helped shape the research, analysis, and manuscript.

## Conflict of Interest

The authors declare that the research was conducted in the absence of any commercial or financial relationships that could be construed as a potential conflict of interest.
