# Supplementary figures and images for: Root Functional Trait and Soil Microbial Coordination: Implications for Soil Respiration in Riparian Agroecosystems
Source: Front Plant Sci. 2021 Jul 8;12:681113. doi: 10.3389/fpls.2021.681113 (PMC8296843; doi:10.3389/fpls.2021.681113)

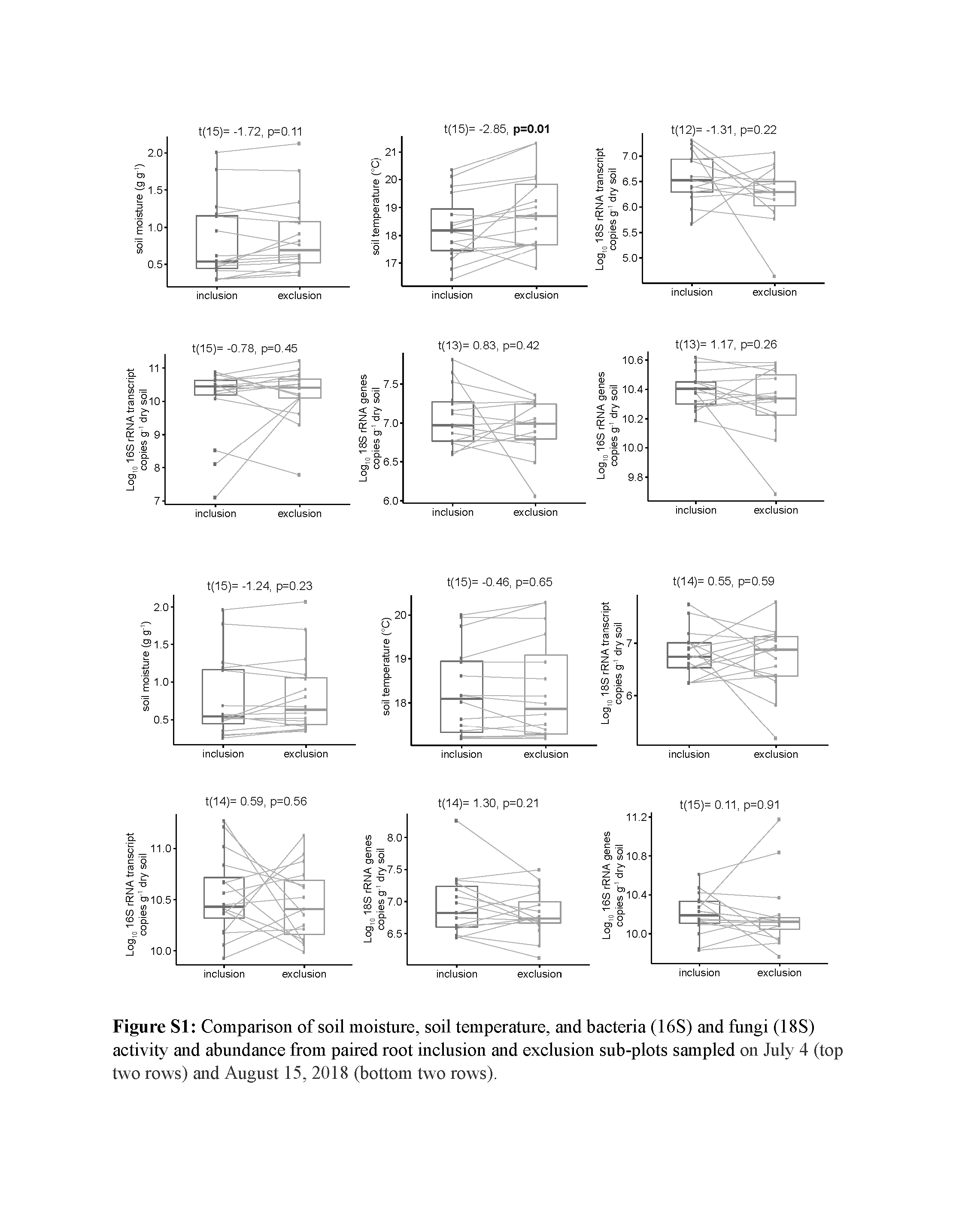

Supplement: Supplementary file 1 [file Image_1.TIF]

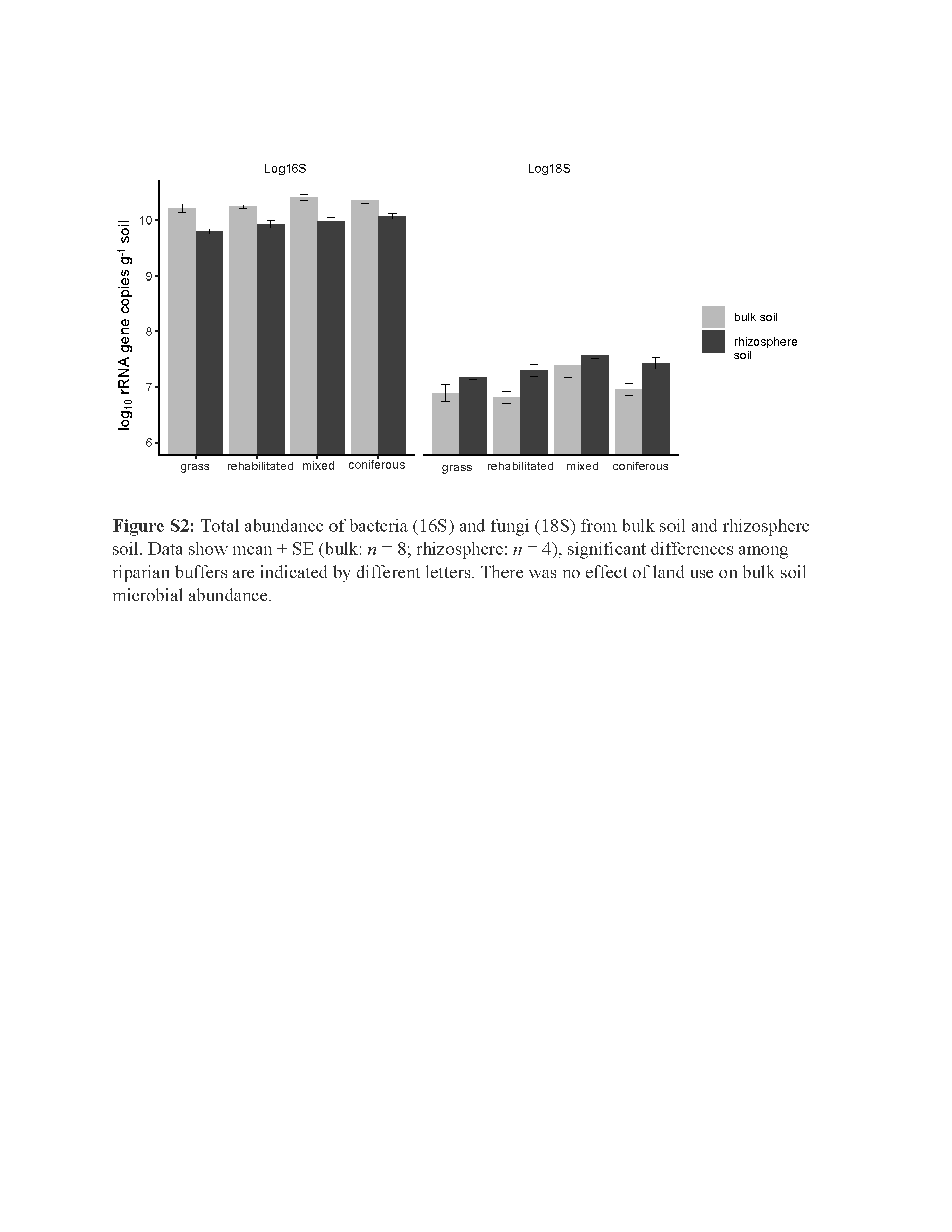

Supplement: Supplementary file 2 [file Image_2.TIFF]
