# Supplementary material for: Root Functional Trait and Soil Microbial Coordination: Implications for Soil Respiration in Riparian Agroecosystems
Source: Front Plant Sci. 2021 Jul 8;12:681113. doi: 10.3389/fpls.2021.681113 (PMC8296843; doi:10.3389/fpls.2021.681113)
Supplement: Supplementary file 3 [file Table_1.DOCX]

**Table S1:** Soil physico-chemical properties (0-10 cm depth) in four riparian buffer types. Values represent the mean ± SE over the experimental period (May – August) except for soil pH and inorganic carbon (at 30 cm depth) which were measured at the start of the experiment.

| Riparian buffer | Soil temp (°C) | Grav. moisture content | Avail. NH_4_^+^ (mg-N kg^-1^) | Avail. NO_3_^-^ (mg-N kg^-1^) | Total N  (g kg^-1^) | Total C  (g kg^-1^) | Soil CN | Inorganic C (g kg^-1^) | Soil pH |
| --- | --- | --- | --- | --- | --- | --- | --- | --- | --- |
| Grass | 16.7 ± 0.5 | 0.47 ± 0.03 | 28.6 ± 4.5 | 33.5 ± 3.0 | 5.5 ± 0.1 | 78.4 ± 1.0 | 14.4 ± 0.2 | 7.8 ± 0.9 | 7.47 ± 0.10 |
| Rehabilitated | 18.6 ± 0.6 | 0.50 ± 0.04 | 21.2 ± 2.8 | 25.7 ± 4.1 | 4.9 ± 0.3 | 71.1 ± 3.3 | 15.0 ± 0.3 | 10.5 ± 0.0 | 7.63 ± 0.03 |
| Coniferous | 15.6 ± 0.5 | 1.07 ± 0.10 | 50.5 ± 4.6 | 17.9 ± 3.1 | 8.3 ± 0.3 | 122.8 ± 4.1 | 14.9 ± 0.3 | 3.2 ± 0.5 | 7.08 ± 0.12 |
| Mixed | 17.0 ± 0.5 | 1.24 ± 0.08 | 70.3 ± 6.9 | 11.2 ± 2.0 | 9.0 ± 0.3 | 134.8 ± 5.3 | 14.9 ± 0.1 | 3.2 ± 1.1 | 7.14 ± 0.10 |
