# Supplementary material for: Root Functional Trait and Soil Microbial Coordination: Implications for Soil Respiration in Riparian Agroecosystems
Source: Front Plant Sci. 2021 Jul 8;12:681113. doi: 10.3389/fpls.2021.681113 (PMC8296843; doi:10.3389/fpls.2021.681113)
Supplement: Supplementary file 4 [file Table_2.docx]

**Table S2:** Summary of soil sampling and analysis of microbial communities in rhizosphere or bulk soil.

| Method step | Soil Regions | |
| --- | --- | --- |
|  | **Rhizosphere soil** | **Bulk soil** |
| Sampling method | Plant roots with closely adhering soil (after shaking) were sampled from root exclusion plots at the start of the experiment and kept on ice until lab | Every two weeks from May-August, soil was sampled at a depth of 0-10 cm four times along a transect, homogenised and 2 g of soil was immediately transferred into pre-weighed sterile tubes containing 3ml of LifeGuard soil preservation solution to stabilise the RNA |
| Processing | Adhering soil and the root samples were vortexed thrice for 30 s each time, and 20 mL from the rhizosphere soil mixture obtained was centrifuged at 5000 r.p.m for 15 min at 5 ˚C as described by Donn et al. (2014) and stored at -20 ˚C | Soil samples stored at -80 ˚C freezer prior to extraction and centrifuged to recover soil pellets for extraction |
| Extraction | DNA was extracted from 0.25 g soil using the PowerSoil DNA Isolation Kit | RNA and DNA were co-extracted using RNeasy PowerSoil™ Total RNA Kit and DNA Elution accessory kit. RNA was reverse transcribed to cDNA and was used for down stream applications |
| Microbial Analysis | qPCR to determine the abundance of targeted genes | qPCR / RT-qPCR to determine abundance/ potential activity of targeted transcripts |
| Sequencing | NONE | Illumina sequencing of 16S rRNA for bacteria, and internal transcribed spacer (ITS) regions for fungi (cDNA) |
