# Supplementary material for: Root Functional Trait and Soil Microbial Coordination: Implications for Soil Respiration in Riparian Agroecosystems
Source: Front Plant Sci. 2021 Jul 8;12:681113. doi: 10.3389/fpls.2021.681113 (PMC8296843; doi:10.3389/fpls.2021.681113)
Supplement: Supplementary file 5 [file Table_3.DOCX]

**Table S3:** Target genes/transcripts and primers used for quantitative polymerase chain reaction (qPCR)

| Transcript/ Genes | Primer name | Primer sequence  (5'-3') | Annealing temperature (^o^C) | Reference |
| --- | --- | --- | --- | --- |
| *16S rRNA* | 338f | ACT CCT ACG GGA GGC AGC AG | 55 | Fierer et al., (2005) |
|  | 518r | ATT ACC GCG GCT GCT GG | 55 |  |
| *18S rRNA* | FF390 | CGATAACGAACGAGACCT | 50 | Vainio and Hantula (2000) |
|  | FR1 | AICCATTCAATCGGTAIT | 50 |  |

***Quantitative real-time PCR for target genes and transcripts***

Briefly, 20 μL qPCR mixture containing 10 μL of Ssofast EvaGreen supermix (Bio-Rad Laboratories, Inc.), 1 μL forward and reverse primer (10 μM), 2 μL DNA or cDNA (1 to 10 ng/μL), and 6 μL of DNase-free water was prepared for each reaction, and qPCR was performed using the thermal cycler (CFX96; Bio-Rad Laboratories, Inc.). Plasmid DNA containing each targeted gene was serially diluted (10^8^–10^1^) and was used to construct standard curves used for the assay. The qPCR thermal cycling conditions and primer details are found in Table S3. Thermal cycling conditions for genes and transcripts were as follows: For bacteria, 16SrRNA gene/transcript, initial denaturation at 98 ^o^C for 2min, followed by 34 cycles of dissociation at 98 ^o^C for 10 s, primer annealing 55 ^o^C for 30 s, extension at 72^o^C for 30 s, and a final extension for 10 min. For Fungi, 18S rRNA genes/transcripts, initial denaturation at 98 ^o^C for 3min, followed by 40 cycles of dissociation at 98 ^o^C for 15 s, primer annealing 50 for 30 s, extension at 72^o^C for 45 s, and a final extension for 10 min. and melt curve analysis (65 °C to 95 °C in 0.5 °C increments for 5 s).
