# Supplementary material for: Root Functional Trait and Soil Microbial Coordination: Implications for Soil Respiration in Riparian Agroecosystems
Source: Front Plant Sci. 2021 Jul 8;12:681113. doi: 10.3389/fpls.2021.681113 (PMC8296843; doi:10.3389/fpls.2021.681113)
Supplement: Supplementary file 6 [file Table_4.DOCX]

**Table S4:** Principal component axes loadings for principal component analysis of root traits analyzed with paired respiration rates (Fig. 2A) and for root traits analyzed with paired microbial abundance data in rhizosphere soil (Fig. 2B).

|  | With specific root respiration | |  | With microbial abundance in rhizosphere | |
| --- | --- | --- | --- | --- | --- |
| Traits | PC1 | PC2 |  | PC1 | PC2 |
| *R_root_* | 1.142^***^ | 1.168^***^ |  | NA | NA |
| SRL | 1.927^***^ | 0.655^**^ |  | 1.072^***^ | 0.388 |
| N_root_ | 1.509^***^ | -1.472^***^ |  | 0.724^*^ | -0.955^***^ |
| D | -1.846^***^ | -0.790^***^ |  | -1.014^***^ | -0.334 |
| C:N_root_ | -1.859^***^ | 0.986^***^ |  | -0.753^**^ | 0.996^***^ |
| 16S | NA | NA |  | -0.864^**^ | -0.258 |
| 18S | NA | NA |  | -0.642^*^ | -0.724^*^ |
| Proportion explained | 0.615 | 0.241 |  | 0.467 | 0.291 |
| Eigenvalue | 3.07 | 1.21 |  | 2.80 | 1.74 |

^***^*p* < 0.001; ^**^*p* < 0.01; ^*^*p* < 0.05
