# Supplementary material for: Root Functional Trait and Soil Microbial Coordination: Implications for Soil Respiration in Riparian Agroecosystems
Source: Front Plant Sci. 2021 Jul 8;12:681113. doi: 10.3389/fpls.2021.681113 (PMC8296843; doi:10.3389/fpls.2021.681113)
Supplement: Supplementary file 7 [file Table_5.DOCX]

**Table S5**. Mean ± SE Shannon diversity, Faith's phylogenetic diversity, Observed ASVs, and Pielou’s evenness in four riparian buffer types inclusive of two sampling dates (*n* = 8).

| Riparian buffer | Shannon | Faith | Observed ASVs | Pielou |
| --- | --- | --- | --- | --- |
| Grass | 8.20 ± 0.05 ab | 33.65 ± 0.85 | 388.5 ± 12.3 ab | 0.96 ± 0.00 ab |
| Rehabilitated | 8.53 ± 0.06 a | 35.75 ± 0.93 | 467.17 ± 19.86 a | 0.97 ± 0.00 a |
| Coniferous | 7.80 ± 0.07 b | 28.99 ± 0.62 | 310.38 ± 12.22 b | 0.95 ± 0.00 b |
| Mixed | 8.28 ± 0.05 ab | 35.78 ± 0.63 | 410 ± 11.92 ab | 0.96 ± 0.00 ab |

Same letters indicate no difference between riparian buffer for each index (Tukey’s HSD)
